# Regulation of Endosomal Sorting and Maturation by ER-Endosome Contact Sites

**DOI:** 10.1177/25152564221106046

**Published:** 2022-06-09

**Authors:** Steve Jean, Sonya Nassari

**Affiliations:** Faculté de médecine et des sciences de la santé, Département d’immunologie et de biologie cellulaire, 12370 Université de Sherbrooke, Sherbrooke, Québec, Canada

**Keywords:** membrane contact sites, endosomes, endoplasmic reticulum, ORP10, TMEM16K, phosphoinositides, OSBP, TMCC1, PIK4IIα/β

## Abstract

Endosomes are a heterogeneous population of intracellular organelles responsible for sorting, recycling, or transporting internalized materials for degradation. Endosomal sorting and maturation are controlled by a complex interplay of regulators, with RAB GTPases and phosphoinositides playing key roles. In this decade, another layer of regulation surfaced with the role played by membrane contact sites between the endoplasmic reticulum (ER) and endosomes. Specific regulators of ER-endosome contact sites or proteins localized at these sites are emerging as modulators of this complex endosomal ballet. In particular, lipid transfer or recruitment of various complexes and enzymes at ER-endosome contact sites play an active role in endosome sorting, scission, and maturation. In this short review, we focus on studies describing ER-endosome contact sites in these three endosomal processes.

## Introduction

**Figure 1.**
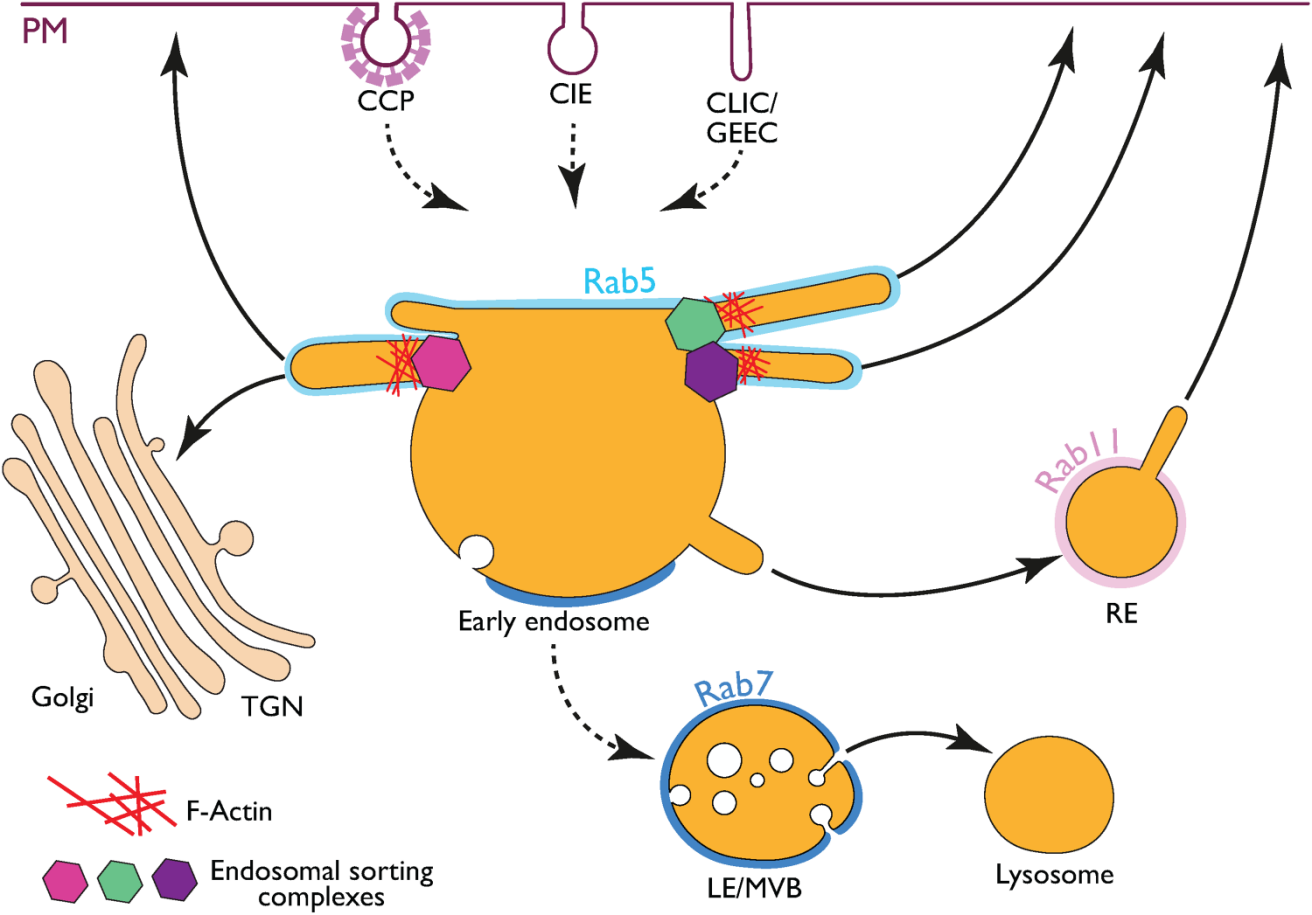
Endosomal sorting complexes and maturation. An ER-endosome MCS independent view. (A) Following clathrin-dependent, clathrin-independent, or CLIC/GEEC endocytosis, endocytic vesicles reach Rab5-enriched early endosomes, where cargos are sorted by various sorting complexes (e.g., retromer, retriever, ccc complex and ESCPE) which cooperate with the WASH complex to direct cargos to a specific location (trans-Golgi network (TGN), plasma membrane (PM), or Rab11 recycling endosomes (RE)), adapted from (Simonetti & Cullen, 2019). Rab7-enriched maturation (degradative) subdomains are also formed on these endosomes, which ultimately mature to RAB7-positive late endosomes/multivesicular bodies (LE/MVB) and fuse with lysosomes.

**Figure 2.**
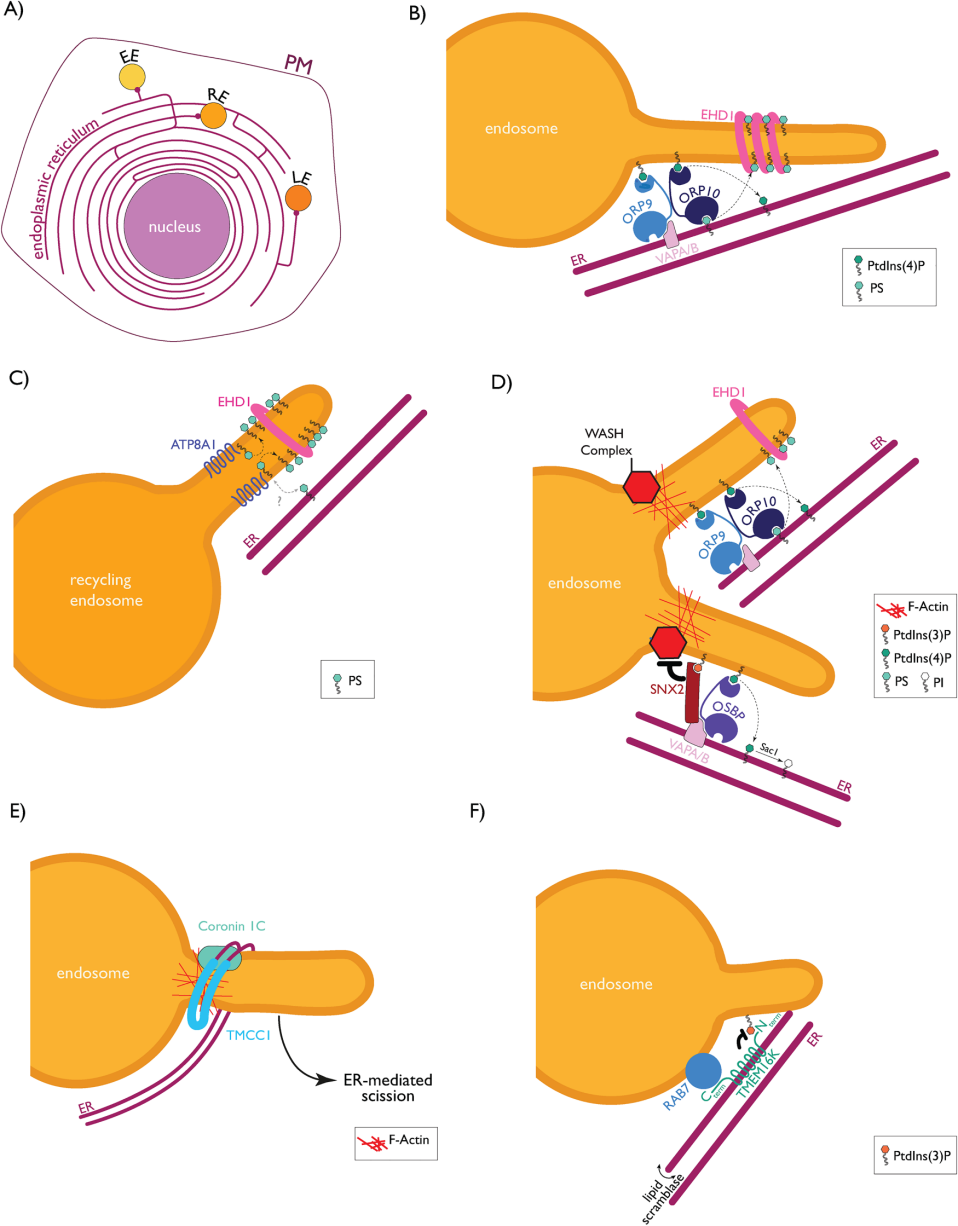
Specific ER-endosome MCSs in cargo sorting and scission. (A) Various ER-endosome MCSs with early endosome (EE), recycling endosome (RE) and late endosome (LE). (B) ORP10 cooperates with ORP9-VAPA/B to mediate PtdIns(4)P and PS counter-transport to regulate endosomal PS level, EHD1 recruitment and tubule scission. Adapted from (Wong et al., 2022) (C) ATP8A1, at recycling endosomes, increases PS cytosolic-leaflet levels and EHD1 recruitment. The grey dotted arrow indicates hypothetical PS transfer between the ER and recycling endosomes. (D) Potential independent or cooperative roles played by PtdIns(4)P, WASH complex, and EHD1 in cargo sorting and tubule scission at ER-endosome MCSs. The lower tubule displays the situation described by Dong et al. (2016), where an OSBP-VAPA/B-SNX2 complex regulates endosomal PtdIns(4)P to affect WASH activity, endosomal actin levels and potentially tubule scission. The upper tubule displays a hypothetical collaboration between ORP9-ORP10, EHD1 and WASH in coordinating cargo sorting (WASH) and endosomal tubule scission (EHD1) (E) TMCC1-coronin 1C interactions at ER-endosome MCSs promote endosomal scission. (F) TMEM16 K binds with its N-terminal domain to PtdIns(3)P, negatively affects endosomal PtdIns(3)P, and generates ER-endosome MCSs through interacting with the C-terminal region of RAB7. ER, endoplasmic reticulum.

Cells constantly exchange molecules with the extracellular environment, and internalized macromolecules transit through sequential vesicular organelles called endosomes. Endosomes are often classified into three populations: early, late, and recycling (Huotari & Helenius, 2011). This classification is mostly related to the presence of specific RAB GTPases and phosphoinositides, as well as to endosomal morphology. Early endosomes receive the bulk of internalized materials (Cullen & Steinberg, 2018). These tubulovesicular organelles harbor heterogeneous subdomains (van der Beek et al., 2022) where various independent sorting events unfold (Figure 1A) (Naslavsky & Caplan, 2018). Sorted cargos are then directed towards the trans-Golgi network (TGN), plasma membrane, or recycling endosomes (Cullen & Steinberg, 2018). As sorting occurs, endosomal subdomains mature, resulting in a late endosome/multivesicular body (Podinovskaia et al., 2021) that ultimately fuses with a lysosome (Figure 1A) (Huotari & Helenius, 2011). Endosomal sorting, mainly regulated by four endosomal sorting complexes, as well as recycling, and maturation were mostly believed to be independent of other organelles. However, ER-endosome membrane contact sites (MCSs) are now emerging as platforms that modulate these events. In this short review, we discuss the roles and involvement of ER-endosome MCSs in endosomal trafficking and maturation (Figure 2A).

## Role of Oxysterol-Binding Protein (OSBP)-Related Proteins (ORPs) at Membrane Contact Sites in Endosomal Scission

Membrane-contact sites are areas where independent organelles are close enough to allow direct communication between them (Prinz et al., 2020). The proximity between organelles permits directed non-vesicular lipid transport and modulation of signaling events, among other processes (Balla et al., 2019; Raiborg et al., 2015b; Wong et al., 2019). ORPs are a class of lipid transfer proteins (LTPs) that mediate lipid exchange at MCSs (Nakatsu & Kawasaki, 2021). They do so by binding and transferring lipids via the OSBP-related domain (ORD), which recognizes various ligands, including oxysterol, cholesterol, phosphoinositides, phosphatidylserine (PS) (Nakatsu & Kawasaki, 2021), and potentially phosphatidylcholine (PC) (D’souza et al., 2020; Tong et al., 2021). Interestingly, ORPs mediate the counter-directional transport of lipids; PtdIns(4)P, in particular, drives the transport of another lipid against a concentration gradient (Mesmin et al., 2013). ER-endosome MCSs are thus important regulators of PtdIns(4)P level in cells, since PtdIns(4)P at endosomes will be transported by ORPs to the ER. Here, the lipid phosphatase SacI will dephosphorylate PtdIns(4)P to yield phosphatidylinositol (PI), ensuring a PtdIns(4)P concentration gradient between endosomes and the ER (Nakatsu & Kawasaki, 2021). ORPs are associated with various ER-organelle MCSs through the concerted action of two determinants: 1) a PH domain that interacts with a specific phosphoinositide and 2) an FFAT motif that binds to the ER-localized (VAMP)-associated protein A (VAPA)/VAPB (Antonny et al., 2018); however, how ORP localization, activity, and functions are regulated is an ongoing question.

Intriguingly, a role in endosomal scission was demonstrated for ORP10, an unconventional ORP that lacks a canonical FFAT motif (Kawasaki et al., 2022). ORP10 was initially identified as a regulator of ER-TGN contact sites (Venditti et al., 2019). The authors demonstrated, through a FRET-FLIM assay measuring ER-TGN contact sites (Venditti et al., 2019), that ORP10 depletion resulted in a decreased occurrence of ER-TGN contact sites. The ability of ORP10 to bind both PtdIns(4)P and PS was required for the occurrence of ER-TGN contact sites (Venditti et al., 2019). An assessment of ORP10 localization through overexpressed fluorescently tagged proteins revealed a predominant Golgi localization in HeLa cells, consistent with a PtdIns(4)P preference for its PH domain (Venditti et al., 2019) and its role in ER-TGN MCS maintenance. However, using 3xHA-tag to generate ORP10-knockin Cos7 cells, Kawasaki et al. (2022) identified a more restricted localization pattern, where ORP10 accumulated in puncta associated with ER tubules. Interestingly, these puncta were colocalized with endosomal proteins, such as RAB5, RAB7, and PI4KIIα, suggesting a potential role for ORP10 in ER-endosome MCSs.

PtdIns(4)P is present in endosome subdomains and most abundant in late endosomes (Baba et al., 2019). This endosomal PtdIns(4)P pool is mostly generated by PI4KIIα/β. Endosomal ORP10 localization is dependent on its PH domain and requires endosomal PtdIns(4)P (Kawasaki et al., 2022). The ER association is contingent on the ORP10 interaction partner ORP9, through a VAPA/B-ORP9 association (Figure 2B). It is worth noting that ORP10 was previously identified to associate with VAPA at the ER by bimolecular fluorescence complementation (BiFC), through an indirect interaction (Weber-Boyvat et al., 2015), alluding to ORP9 as a general modulator of ORP10 ER recruitment. Both *in vitro* and *in vivo* experiments show that ORP10 mediates PtdIns(4)P and PS counter-transport in the ER-endosome MCS to increase endosomal PS levels (Kawasaki et al., 2022). Deletion of ORP10 or ORP9 decreased endosomal PS, suggesting that the two proteins are both needed for PS transport. Given that cytoplasmic expression of lactadherin C2 domain detects cytosolic-facing PS (Lee et al., 2015), any observed PS decrease likely occurred on the endosome cytosolic leaflet. Contrary to expectations, ORP10 deletion did not affect PtdIns(4)P levels, whereas loss of ORP9 increased it. A possible hypothesis is that ORP9 may have provided some compensatory mechanism. Although not tested, ORP11, a close ORP10 paralogue could also have compensated for ORP10 loss.

Considering the endosomal localization of ORP10, Kawasaki et al. (2022) analyzed whether differences in endosomal PS levels affected trafficking events, revealing that retrograde transport of cation-independent mannose 6-phosphate receptor (CI-MPR) was reduced in ORP10-deleted cells. These authors also assessed EHD1 recruitment in ORP10 KO cells, given that PS recruits EHD1 (Lee et al., 2015) a well-known regulator of tubulovesicular scission (Deo et al., 2018; Naslavsky & Caplan, 2011). They found that endosomal EHD1 recruitment decreased with lower PS levels, leading to slower kinetics of SNX1-labelled tubule fission (Kawasaki et al., 2022). Additionally, they showed that ORP10 activity in retrograde trafficking required the ORD domain, implying that PtdIns(4)P and PS counter-transport was essential; while endosomal sorting complexes were unaffected. Altogether, this manuscript provided evidence that ORP10 at ER-endosome contact sites, is an important modulator of PS accumulation and endosomal fission (Figure 2B).

In line with the role of PS in EHD1 recruitment, Lee et al. (2015) demonstrated in Cos1 cells that ATP8A1, a P4-ATPase that translocates PS to the cytosolic leaflet of membranes is required for recycling endosome tubule scission. Importantly, this study showed that ATP8A1 is highly localized to recycling endosomes and is required to enrich PS on the cytosolic side of these endosomes, resulting in EHD1 recruitment and tubule scission (Figure 2C). Recycling endosomes are also far more enriched with PS than early or late endosomes. Together with findings from Kawasaki et al. (2022), these data suggest that PS is essential for EHD1 recruitment and cargo sorting across multiple endosome types. We can also speculate that ORP10 and ATP8A1 mediate endosomal scission through a shared pathway that allows high PS levels at specific endosomal subdomains. An intriguing experiment will be to investigate combined deletions of these regulators or whether overexpression of one could rescue the loss of the other. Moreover, these ER-endosome MCSs may allow for the recruitment of other endosomal sorting regulators, such as the WASH complex, to potentiate sorting of specific cargo classes.

## Phosphoinositides in ER-Endosome MCS

Phosphoinositides are well-described regulators of membrane trafficking (Jean & Kiger, 2012) and subtle changes in their levels affect a large array of cellular functions (Hammond & Burke, 2020). Further highlighting a link between PtdIns(4)P and other endosomal phosphoinositides in endosomal sorting, it had been observed that a transition between PtdIns(3)P and PtdIns(4)P governed by the myotubularin MTM and PI4KIIα was required for endocytic recycling of integrins and transferrin receptor (Ketel et al., 2016). Interestingly, this conversion mechanism requires PtdIns(4)P at RAB11 recycling endosomes to allow for appropriate exocyst recruitment. Because ER-endosome MCSs regulate PtdIns(4)P (Dong et al., 2016; Kawasaki et al., 2022; Petkovic et al., 2020) and recycling endosomes have high PS concentrations (Lee et al., 2015), it is tempting to speculate that ORP10-mediated counter-transport at PI4KIIα endosomes coordinates with other endosomal PIP phosphatases and kinases to fine-tune tubule scission with sorting events and tubule extensions. This process would likely affect a larger spectrum of cargos than only the CI-MPR previously described (Kawasaki et al., 2022).

Another interesting link between endosomal phosphoinositides and ER-endosome contact sites is the effect of OSBP- and VAPA/VAPB-mediated MCS on PtdIns(4)P regulation (Dong et al., 2016). The SNX-BAR protein SNX2 interacts with PtdIns(3)P in the early endosome (Dong et al., 2016), and SNX2 overexpression in COS-7 cells leads to VAPA/B accumulation at SNX2-positive endosomes. Importantly, the SNX2-mediated VAPA/B accumulation requires endosomal PtdIns(3)P, suggesting that SNX2 interacts with VAPA/B at ER-endosome MCSs (Dong et al., 2016). At these MCSs, OSBP transfers PtdIns(4)P to the ER, where it is dephosphorylated to PI by Sac1 phosphatase (Dong et al., 2016) (Figure 2D). In experiments with HeLa cells, OSBP or VAPA/B deletion increased endosomal PtdIns(4)P levels. In contrast to the ORP10 phenotype (Kawasaki et al., 2022), endosomal PtdIns(4)P accumulation leads to aberrant WASH recruitment and increased actin polymerization in endosomes (Dong et al., 2016). Aberrant WASH activity then causes defects in CI-MPR trafficking. Although the exact mechanism is unclear, increased WASH recruitment from PtdIns(4)P accumulation is dependent on PI4KIIα/β, meaning the latter are key regulators of endosomal sorting and fission. Notably, cortactin-binding PtdIns(3,5)P_2_ reduces actin in RAB7 endosomes (Hong et al., 2015). Moreover, WASH depletion leads to increased endosomal tubulation (Derivery et al., 2009). These findings highlight a potential parallel pathway to the one involving ORP10, given that OSBP-VAPA/B ER-endosome MCSs are more prevalent in RAB7-positive endosomes. In this scenario, phosphoinositide (PtdIns(3)P and PtdIns(4)P) regulation controls endosomal scission through WASH recruitment and activity, whereas PS transport coupled with EHD1 recruitment influences endosomal fission of different cargos or towards different destinations (Figure 2D, lower tubule). Alternatively, WASH recruitment could occur at ORP10-regulated ER-endosome MCSs to regulate cargo sorting more than tubule fission (Figure 2D, higher tubule). In support of this possibility, WASH-induced actin polymerization reduces the diffusion of sequestered cargo for efficient scission by EHD1 (Simonetti & Cullen, 2019). Finally, it is worth mentioning that mutations in the spastin, strumpellin, or REEP1 genes cause hereditary spastic paraplegia (HSP). Spastin is present at ER-endosome contact sites in neurons and influences endosomal tubule fission (Allison et al., 2017), potentially acting together or in parallel with ORP10. These examples denote strong links between endosomal phosphoinositides and ER-endosome contact sites.

## Direct Regulation of ER-Endosome MCS Formation in Cargo Sorting

The ER establishes numerous connections with other intracellular organelles through MCSs (Valm et al., 2017). These MCSs have profound implications for trafficking events, as already summarized; however, the direct effects of the tested proteins on MCS formation and maintenance were not investigated in previous studies. Modulation of ER-endosome contact sites was found to affect endosomal carrier fission through recruiting the WASH complex to endosomal tubules (Rowland et al., 2014). This process is dependent on the TMCC class of ER-anchored proteins (Hoyer et al., 2018). TMCC1 is recruited to tubule bud sites by coronin 1C, an actin regulatory protein present in RAB7 endosomes. Hence, when either TMCC1 or coronin 1C is reduced, both ER-endosome MCSs and CI-MPR trafficking decrease (Figure 2E). This change is likely independent of WASH-mediated actin dynamics, given that WASH/FAM21 deletion did not affect ER recruitment or fission of endosomal tubules. Therefore, the TMCC1/coronin 1C-regulated ER-endosome MCS would directly affect endosomal tubule scission, while WASH recruitment would most likely control cargo sorting, rather than the fission event.

The active sorting of signaling receptors into intraluminal vesicles (ILVs) occurs throughout the endosomal maturation stage, and relies, at least partly, on the ER-endosome contact sites. Specifically, EGFR downregulation and lysosomal degradation upon activation require EGFR incorporation into the ILVs of multivesicular bodies (Tomas et al., 2014). A specific subset of ER-endosome contact sites, scaffolded by Annexin A1, affects EGFR signaling (Eden et al., 2016). Interestingly, the overexpression of Annexin A1 promoted the occurrence of ER-endosome MCSs. This ability of Annexin A1 to modulate ER-endosome MCSs is directly influenced by the EGFR: a phosphomimetic mutant of Annexin A1 at the EGFR phosphorylation site, increases the extent of ER-endosome MCSs compared to wild-type cells, while a non-phosphorylatable mutant does not affect EGFR-driven MCSs (Eden et al., 2016). Moreover, the ER-localized tyrosine phosphatase, PTP1B, affects the EGFR at ER-endosome MCSs (Eden et al., 2010), and the EGFR-specific ER-endosome MCSs, scaffolded by Annexin A1, serve as action sites on the EGFR for PTP1B (Wong et al., 2018). These instances highlight that in addition to scission events, ER-endosome MCSs also affect receptor sorting into ILVs.

## TMEM16K in ER-Endosome MCS and Endosomal Sorting

In addition to the above examples, a connection between phosphoinositides, RAB GTPases, and ER-endosome MCSs modulated by TMEM16 K, an ER-localized lipid scramblase, has been uncovered (Petkovic et al., 2020). Mutations in TMEM16 K are causative of spinocerebellar ataxia (SCAR10) (Vermeer et al., 2010), and TMEM16 K is localized in the ER of U2OS cells (Petkovic et al., 2020), in addition to other cells (Kramer & Hawley, 2003; Maass et al., 2009). Using a BioID approach, the authors identified multiple links between TMEM16 K, the ER, and endolysosomal proteins (Petkovic et al., 2020). Comparison of wild-type and TMEM16 K KO mouse embryonic fibroblasts highlighted deficient anterograde trafficking of CI-MPR and CtxB in TMEM16 K mutant cells, while the anterograde secretory pathway was unaffected. Significantly, TMEM16 K was in close proximity to VAPA/B, SNX1, SNX2, and RAB7A, and the RAB7A/TMEM16 K interaction was demonstrated to form endosome-ER contact sites. The RAB7A/TMEM16 K interaction was mediated by the C-terminal region of TMEM16 K. A PIP-strip overlay assay identified binding of the TMEM16 K N-terminus to endosomal phosphoinositides PtdIns(3)P, PtdIns(4)P, and PtdIns(3,5)P_2_, implying that TMEM16 K links ER to endosomes via multivalent interactions. However, TMEM16 K deletion did not affect the number of ER-endosome MCSs, nor did it affect endosomal PtdIns(4)P levels, suggesting that its effects on endosomal sorting differed from those of ORP10 (Kawasaki et al., 2022). Accordingly, TMEM16 K deletion was associated with increased PtdIns(3)P levels, indicating a potential problem in phosphoinositide conversion rather than PtdIns(4)P accumulation.

The need for TMEM16 K scramblase activity suggests that transfer between membrane leaflets of specific phospholipids is required for efficient endosomal sorting (Figure 2F). Because the ORP10 paralog ORP11 was identified in the TMEM16 K BioID dataset (Petkovic et al., 2020), TMEM16 K could potentially act in the same pathway as ORP10 and ATP8A1 to ensure an appropriate PS concentration on endosome cytosolic leaflets.

## MCS in Endosomal Maturation

**Figure 3.**
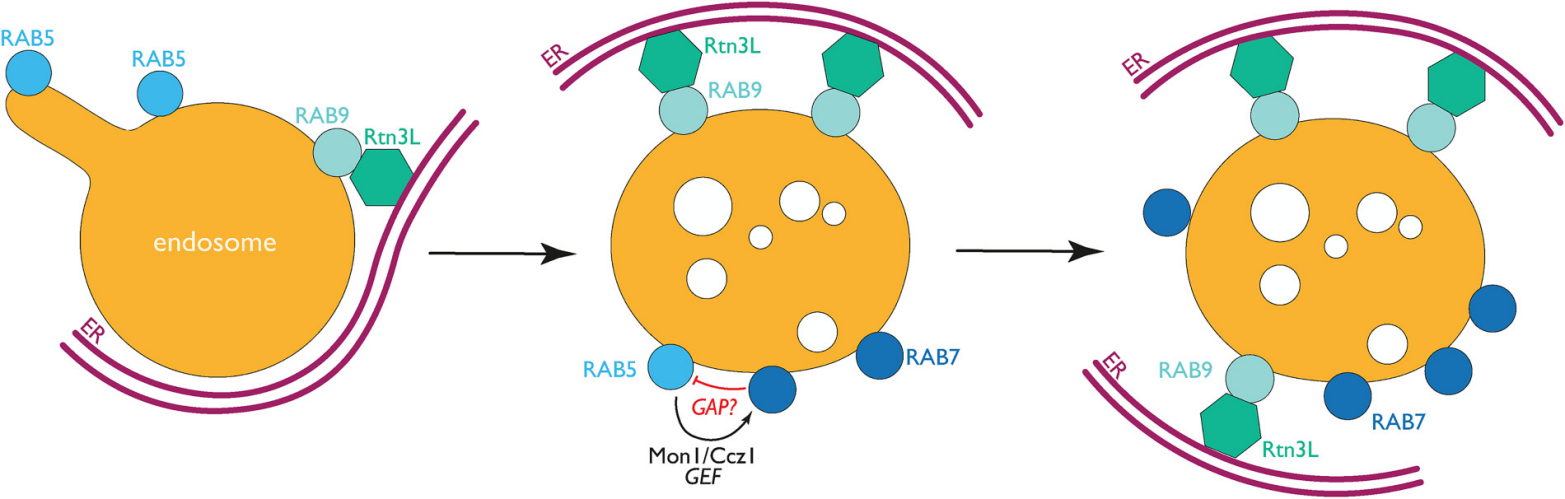
Rtn3L and RAB9 mediation of ER-endosome MCSs affects endosomal maturation. Rtn3L/ER-related binding to RAB9 at late endosomes favors the RAB5 to RAB7 conversion of endosomes. The RAB5 effector Mon1/Ccz1, a RAB7 GEF, activates RAB7 following RAB5 binding and regulates the conversion of RAB5 to RAB7 (Langemeyer et al., 2020). Albeit GAPs have been described for both RAB5 (i.e. RN-Tre, RABGAP-5) and RAB7 (i.e. TBC1D15, TBC1D5) a RAB5 GAP that binds RAB7 as an effector has only been characterized in the context of macropinocytosis in *Dictyostelium* (PripA-TbcrA) (Tu et al., 2022), while the MON1 binding protein, TBC1D18, potentially acts as a RAB5 GAP in endosomal maturation (Hiragi et al., 2021). ER, endoplasmic reticulum.

The number of ER-endosome contact sites increases as endosomes mature (Friedman et al., 2013), observable through the conversion of Rab5-positive endosomes to Rab7. This maturation-related switch is mediated in part by the MON1A/B-CCZ complex, which removes Rab5 GEF while concomitantly activating Rab7 (Langemeyer et al., 2020; Poteryaev et al., 2010; Rink et al., 2005). A study tested whether the increase in ER-late endosome contact sites is coincidental or plays a role in endosomal maturation (Wu & Voeltz, 2021). Mechanistically, it was demonstrated that the ER-resident protein Rtn3L was recruited to ER-endosome MCSs by Rab9a, which is found at late endosomes along with Rab7 (Wu & Voeltz, 2021). Depletion of either Rab9 or Rtn3L impaired endosomal maturation, with depleted cells showing a significant increase in the percentage of Rab5/Rab7 double-positive endosomes, suggesting defective endosomal conversion. Depletion also affected cargo sorting via reducing CI-MPR localization at the TGN and decreasing epidermal growth factor receptor (EGFR) degradation after stimulation. Altogether, these findings illustrate that Rtn3L and Rab9 affect endosome maturation and cargo sorting at ER-endosome MCSs (Figure 3A). It will be interesting to test if these MCSs could participate in the recruitment of known endosomal conversion regulators like the MON1A/B-CCZ complex.

The frequency and duration of ER-endosome contact were found to be independent of microtubules (Rowland et al., 2014). Nonetheless, in a different context, ER-endosome contact affects late endosome movement during neurite outgrowth or in response to cholesterol levels. Interestingly, in neurons, the ER-anchored protein protrudin can interact with late endosomal Rab7 and PtdIns(3)P to form ER-endosome MCSs (Raiborg et al., 2015a). Given that protrudin interacts with kinesin motors, these ER-endosome MCSs would allow the handoff of kinesin complexes to Rab7/FYCO1 complexes. This results in the directed displacement of late endosomes, facilitating neurite outgrowth. These protrudin/FYCO1 handoffs happen stochastically as late endosomes move.

The intracellular localization of late endosomes also appears to be regulated by ORP1L-controlled ER-endosome MCSs, in response to cholesterol levels (Rocha et al., 2009). At high levels, the ORD domain of ORP1L binds to lysosomal cholesterol and forms a complex with Rab7/RILP, which interacts with the dynein/dynactin complex to modulate minus-end transport. When cholesterol is low, however, ORP1L does not bind to it, allowing the ORP1L FFAT motif to interact with VAPA/B and decrease late endosomal movement.

Although ER-endosome contact sites affect multiple biological functions, in most cases, deletion of localized proteins does not affect the contact sites. Hence, defining the minimal or interacting factors regulating ER-endosome MCS is important. A study identified a novel ER-endosome regulator that mediates three-way contact sites between the ER endosome and mitochondria (Elbaz-Alon et al., 2020). The ER-transmembrane protein PDZD8 interacts with protrudin in the ER, and both proteins interact with endosomal Rab7 to scaffold ER-endosome-mitochondria contact sites. The functional role of these MCSs is unknown. However, overexpressing the Rab5-to-Rab7 conversion regulator WDR1 (Liu et al., 2016) with PDZD8 strongly increases the number of ER-late endosome contact sites, suggesting a potential role in endosomal maturation.

## Conclusions

The various manuscripts highlighted illustrate a strong influence of ER-endosome MCSs on endosomal sorting and maturation. Significantly, many proteins visit these interaction platforms, with PtdIns(4)P and RABs being central players in various ER-endosome MCSs.

While yeasts have been instrumental in defining the role of various types of MCS *in vivo*, most studies to date on ER-endosome MCSs have been performed in only a few transformed cell lines. Hence, future research should aim to better define the impact of ER-endosome MCSs in multicellular organisms. Given the conservation of these genes in model organisms and the ease of performing knock-ins by CRISPR/Cas9, it will be interesting of generating point mutants unable to localize at or scaffold ER-endosome MCSs, instead of full knockdown, and to evaluate how these contact sites affect the organism. Future *in vivo* projects will prove very informative in defining the exact roles of ER-endosome MCSs in human disease.

Finally, the known roles of ER-endosome MCSs in endosomal sorting and maturation have largely been derived from studying CI-MPR or EGFR trafficking. Because endosomal sorting complexes regulate a large array of transmembrane proteins, we should expand studies to more cargo so that we can decipher how these various ER-endosome contacts overlap at the level of cargos. Again, using *in vivo* models might highlight phenotypes that will lead to specific cargos, thus expanding the role of ER-endosome MCSs. Many discoveries have provided insight that hints at this expanded role, clarifying the precise biological functions and organismal requirements of ER-endosome MCSs, as well as their interplay with other relevant endosomal processes, will be truly interesting.
